# Author Correction: Endogenous FGF21-signaling controls paradoxical obesity resistance of UCP1-deficient mice

**DOI:** 10.1038/s41467-021-22119-x

**Published:** 2021-03-16

**Authors:** Susanne Keipert, Dominik Lutter, Bjoern O. Schroeder, Daniel Brandt, Marcus Ståhlman, Thomas Schwarzmayr, Elisabeth Graf, Helmut Fuchs, Martin Hrabe de Angelis, Matthias H. Tschöp, Jan Rozman, Martin Jastroch

**Affiliations:** 1 Institute for Diabetes and Obesity, Helmholtz Diabetes Center, Helmholtz Zentrum München, German Research Center for Environmental Health (GmbH), 85764 Neuherberg, Germany; 2 German Center for Diabetes Research (DZD), 85764 Neuherberg, Germany; 3 Department of Molecular Biosciences, The Wenner-Gren Institute, Stockholm University, 106 91 Stockholm, Sweden; 4 Wallenberg Laboratory and Sahlgrenska Center for Cardiovascular and Metabolic Research, Institute of Medicine, University of Gothenburg, 413 45 Göteborg, Sweden; 5 Institute of Human Genetics, Helmholtz Zentrum München, German Research Center for Environmental Health (GmbH), 85764 Neuherberg, Germany; 6 German Mouse Clinic, Institute of Experimental Genetics, Helmholtz Zentrum München, German Research Center for Environmental Health (GmbH), 85764 Neuherberg, Germany; 7 Chair of Experimental Genetics, School of Life Science, Weihenstephan, Technische Universität München, 85354 Freising, Germany; 8 Division of Metabolic Diseases, Department of Medicine, Technische Universität, Munich, Germany; 9 Present Address: Laboratory for Molecular Infection Medicine Sweden (MIMS) and Department of Molecular Biology, Umeå University, 901 87 Umeå, Sweden; 10 Present Address: Czech Centre for Phenogenomics, Institute of Molecular Genetics of the Czech Academy of Sciences BIOCEV, 252 50 Vestec, Czech Republic

**Keywords:** Physiology, Metabolism

Correction to: *Nature Communications* 10.1038/s41467-019-14069-2, published online 31 January 2020.

The original version of the Peer Review File associated with this Article was updated after publication to redact two figures in the interest of confidentiality.

## Supplementary information

Peer Review File

