# Supplementary material for: Endogenous FGF21-signaling controls paradoxical obesity resistance of UCP1-deficient mice
Source: Nat Commun. 2020 Jan 31;11:624. doi: 10.1038/s41467-019-14069-2 (PMC6994690; doi:10.1038/s41467-019-14069-2)
Supplement: Supplementary file 1 — Supplementary Information [file 41467_2019_14069_MOESM1_ESM.pdf]

Supplemental material

Endogenous FGF21-signaling controls paradoxical obesity resistance of UCP1-deficient mice

Susanne Keipert[1,2,3#], Dominik Lutter[1,2], Bjoern O. Schroeder[4], Daniel Brandt[1,2], Marcus Ståhlman[4], Thomas Schwarzmayr[5], Elisabeth Graf[5], Helmut Fuchs[6], Martin Hrabe de Angelis[2,6, 7], Matthias H. Tschöp[1,2,8], Jan Rozman[2,6], Martin Jastroch[1,2,3 #]

Supplemental Figure 1

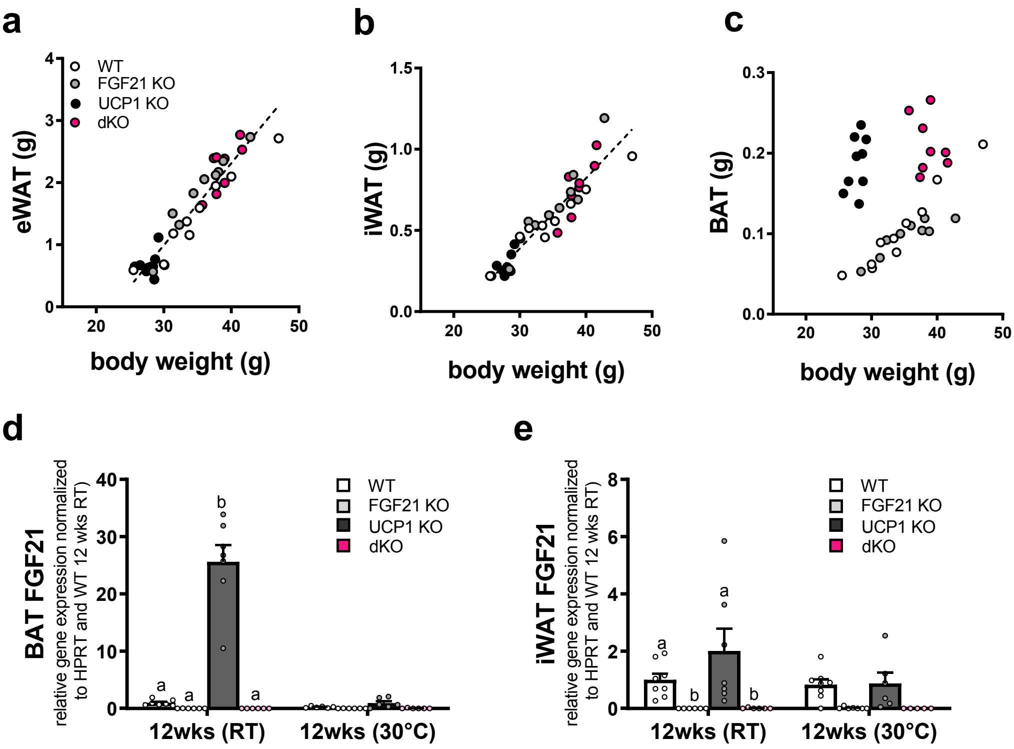

Figure S1 (related to figure 2): WT, FGF21 KO, UCP1 KO and UCP1/FGF21 dKO mice were weaned and raised at 30°C. At the age of 10-12 wks, animals were transferred from 30°C to 23°C, and the diet was changed to high fat diet (HFD) for 12 weeks. Body weights (x-axis) were plotted against tissue weights of (a) (e)pididymal, (b) (i)nguinal WAT and (c) BAT. FGF21 gene expression in (d) BAT and (e) iWAT after 12 weeks of HFD at 23°C (room temperature; RT) or 30°C. a-c: n = 10/9/9/8 WT/ FGF21 KO/ UCP1 KO/ dKO; d-e: n = 7/6/7/6 – 6/6/6/6 WT/ FGF21 KO/ UCP1 KO/ dKO 23°C - WT/ FGF21 KO/ UCP1 KO/ dKO 30°C. Data (d-e) were analyzed by one-way ANOVA followed by Bonferroni test and presented as mean + SEM with different letters indicating significant differences ($P < 0.05$).

Supplemental Figure 2

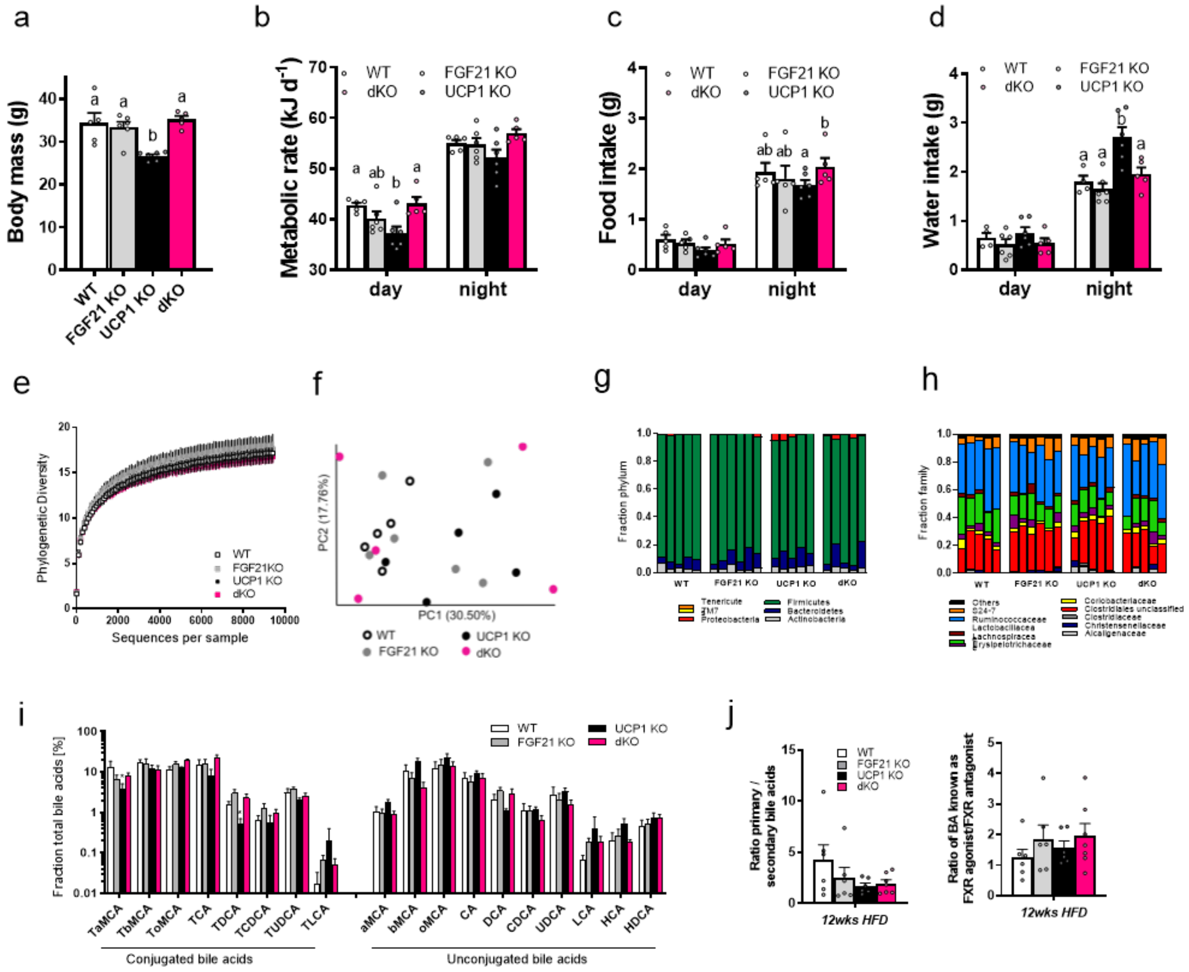

Figure S2 (related to figure 3): WT, FGF21 KO, UCP1 KO and UCP1/FGF21 dKO mice were weaned and raised at 30°C. At the age of 10-12 wks, animals were transferred from 30°C to 23°C, and to high fat diet (HFD). After 9 wks HFD feeding the animals were placed into an indirect calorimetry system. (a) Mean body weight, (b) metabolic rate, (c) food intake and (d) water intake were measured. After 12 wks HFD feeding, feces and serum were collected. (e) Alpha rarefaction plot displays species richness dependent on sampling depth (max depth = 9,410 sequences per sample). (f) Principal coordinate analysis (PCoA, weighted UniFrac) displays β-diversity of the gut microbial community. The percentage of the variation explained by the plotted principal coordinates is indicated at the axis labels. Each dot represents a caecal community. Relative abundance at (g) phylum and (h) family level in caecal community of mice. Only relative abundances > 1% are displayed. (i) Plasma bile acid (BA) profile and (j) ratio of primary to secondary BAs and ratio of BAs, known as FXR agonist/FXR antagonist. a, b: n = 5/6/6/5 WT/ FGF21 KO/ UCP1 KO/ dKO; c: n = 5/5/6/5; d: n = 4/6/6/5; i: n = 6/6/6/6; j, i: n = 6/6/6/7. Data (a-d) were analyzed by one-way ANOVA followed by Bonferroni test and presented as mean + SEM with different letters indicating significant differences ($P < 0.05$). Data are presented as mean ± SD (e) or mean + SEM (i-j) with significant differences shown as *$P < 0.05$.

Supplemental Figure 3

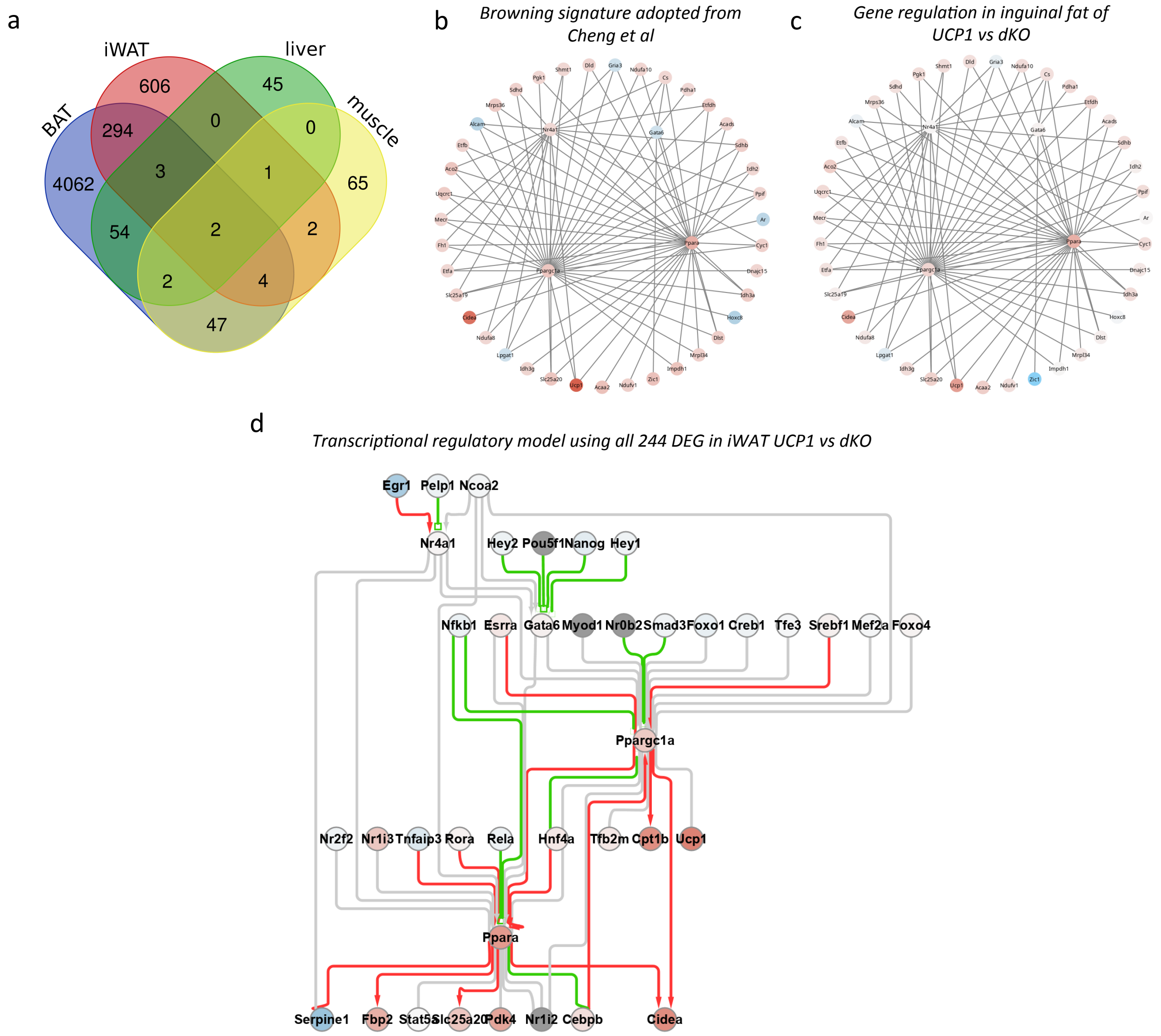

Figure S3 (related to figure 5): WT, FGF21 KO, UCP1 KO and UCP1/FGF21 dKO mice were weaned and raised at 30°C. At the age of 10-12 wks, animals were transferred from 30°C to 23°C, and diet was changed to high fat diet (HFD) for 3 wks. (a) Venn analysis of significantly expressed transcripts of all tissues (adjusted p-value < 0.01). (b) Model predicting regulation of browning adopted from Cheng et al. 2018, Cell Reports; and (c) model applied to regulated genes of UCP1 KO vs dKO in iWAT. Node colors correspond to the log2 FC of UCP1 KO vs dKO in iWAT. (d) Main regulators of browning were taken from S4a and S4b (Gata6, Ppargc1a, Nr4a1, Ppara). TRRUST (https://www.grnpedia.org/trrust/) database was used to create a transcriptional regulatory model using all 244 DEG in iWAT, UCP1 KO vs dKO (adjusted p-value < 0.001). Only direct interactions with the known browning factors were selected. Network visualization was performed using cytoscape. Node colors refer to log2 FC (red = upregulation, blue = downregulation). Genes in gray were added by the TRRUST database. Edge colors refer to regulationtype: red activation, green = repression, gray = unknown (n=5/5/5/5 WT/ FGF21 KO/ UCP1 KO/ dKO).

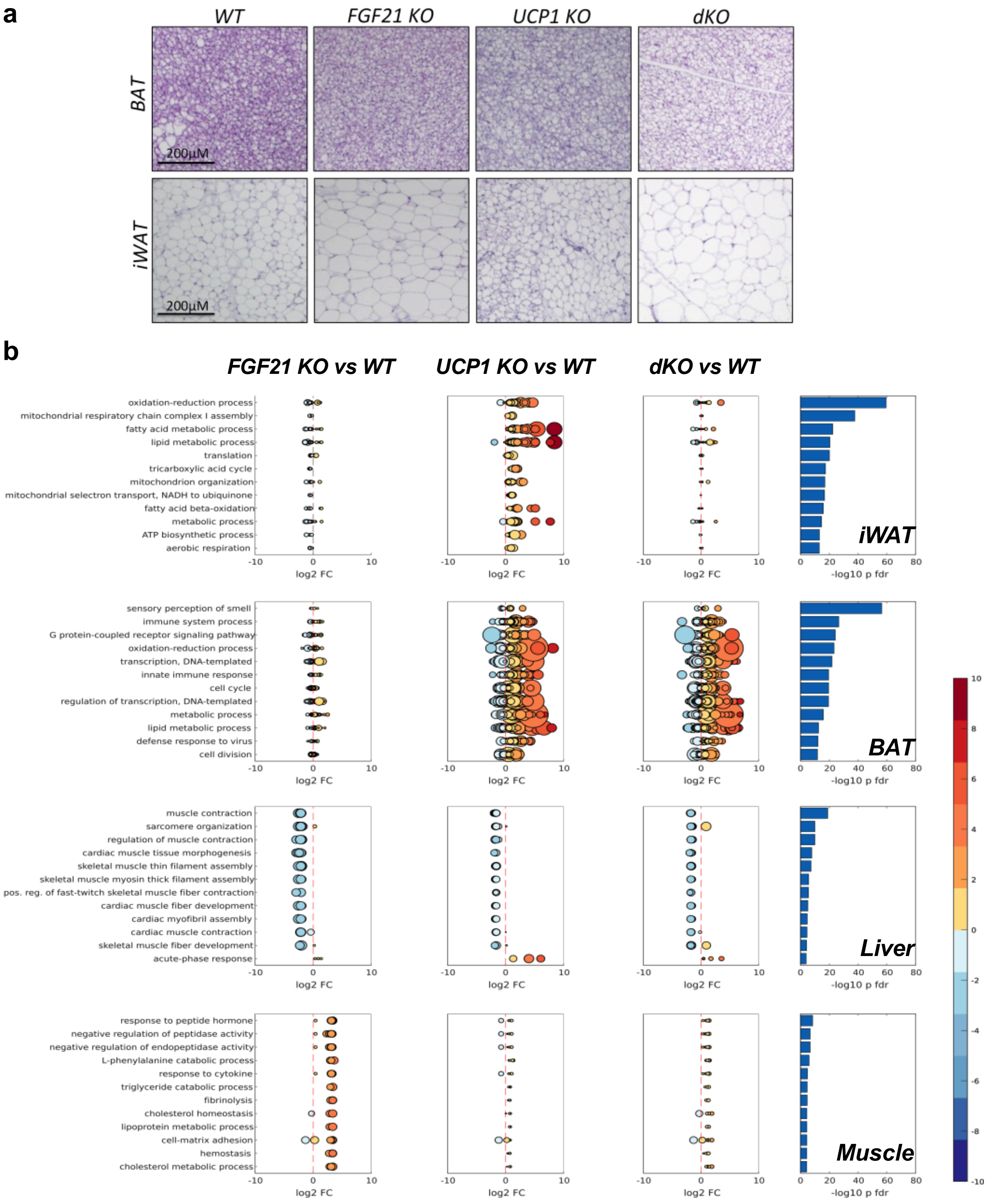

Figure S4 (related to figure 5): WT, FGF21 KO, UCP1 KO and UCP1/FGF21 dKO mice were weaned and raised at 30°C. At the age of 10-12 wks, animals were transferred from 30°C to 23°C and high fat diet (HFD). (a) Representative picture of the morphology in iWAT and BAT after 12 weeks of HFD feeding. (b) GO enrichment analysis of significantly regulated genes in iWAT, BAT, liver and muscle of WT vs FGF21 KO; WT vs UCP1 KO and WT vs dKO after 3 wks HFD. The top 12 regulated pathways are shown. Dot plots display expression of single genes related to the enriched pathways. Dot colors refer to log2 FoldChange, while dot size refers to -log10 significance of regulation (n=5/5/5/5 WT/FGF21 KO/UCP1 KO/ dKO).

Supplemental Table1
List of urine metabolites (AbsoluteIDQ p180 Kit - Biocrates Life Science AG)

AbsoluteIDQ p180 Kit (Biocrates Life Science AG); all data normalized to Creatinine ([µmol/mmol Creatinine])

		WT		FGF21 KO		dKO		UCP1 KO	
		Mean	SE	Mean	SE	Mean	SE	Mean	SE
Aminoacids	**Gly**	134,1	5,9	146,7	4,1	138,9	5,5	124,2	6,3
Aminoacids	**Gln**	81,6	4,8	87,7	17,1	79,6	4,2	100,3	13,4
Aminoacids	**Ala**	44,2	1,3	37,94	1,663	46,2	1,7	58,8	2,775
Aminoacids	**Thr**	44,0	2,5	29,2	1,4	41,3	2,4	64,7	4,2
Aminoacids	**Met**	29,4	2,5	23,0	6,2	24,6	2,9	37,04	4,435
Aminoacids	**Glu**	22,8	2,5	22,7	2,2	24,9	1,5	25,1	1,9
Aminoacids	**Leu**	22,1	1,1	23,7	1,7	23,2	1,1	26,9	1,8
Aminoacids	**Ser**	18,7	0,8	17,7	1,8	18,2	0,8	20,0	2,2
Aminoacids	**Tyr**	17,4	0,8	19,3	4,6	15,8	0,8	27,8	5,2
Aminoacids	**Lys**	16,9	0,9	15,6	1,3	17,0	1,0	18,5	2,0
Aminoacids	**Pro**	15,0	1,0	13,8	2,3	14,6	0,6	21,9	2,9
Aminoacids	**Asn**	14,9	0,5	13,9	1,0	15,5	0,4	15,2	1,3
Aminoacids	**His**	11,6	0,5	11,2	1,7	12,9	0,5	15,1	1,6
Aminoacids	**Val**	11,4	0,6	11,4	0,9	12,2	0,7	13,9	1,2
Aminoacids	**Arg**	9,7	0,5	8,5	0,9	9,5	0,6	8,4	0,9
Aminoacids	**Phe**	9,4	0,3	10,3	1,5	10,0	0,6	13,7	1,4
Aminoacids	**Ile**	8,0	0,4	8,7	0,7	9,0	0,6	11,5	0,7
Aminoacids	**Orn**	5,3	0,2	5,0	0,5	5,3	0,4	6,2	0,5
Aminoacids	**Asp**	4,4	0,2	4,1	0,3	4,1	0,2	5,0	0,5
Aminoacids	**Trp**	2,2	0,1	2,4	0,3	2,4	0,2	3,1	0,3
Aminoacids	**Cit**	2,0	0,1	2,1	0,3	2,0	0,1	2,4	0,2
Biogenic Amins	**Creatinine**	1000,0	0,0	1000,0	0,0	1000,0	0,0	1000,0	0,0
Biogenic Amins	**Putrescine**	44,4	3,2	37,9	8,1	37,2	2,6	50,9	4,2
Biogenic Amins	**Met-SO**	29,4	1,4	23,8	2,5	29,7	2,4	33,6	3,4
Biogenic Amins	**alpha-AAA**	22,9	4,7	3,9	0,9	9,9	2,3	26,2	8,8
Biogenic Amins	**ADMA**	12,8	0,5	11,4	1,2	13,5	0,4	16,3	0,8
Biogenic Amins	**Carnosine**	6,7	0,6	5,8	1,3	4,9	0,6	6,4	0,8
Biogenic Amins	**t4-OH-Pro**	5,0	0,4	4,3	0,4	4,4	0,6	5,4	0,8
Biogenic Amins	**Spermidine**	3,6	0,2	2,7	0,1	3,1	0,4	2,3	0,1
Biogenic Amins	**Sarcosine**	3,2	0,3	3,6	0,7	4,0	0,4	5,8	1,0
Biogenic Amins	**Serotonin**	0,9	0,0	0,8	0,1	1,0	0,0	1,1	0,0
Biogenic Amins	**Dopamine**	0,78	0,08	0,84	0,04	1,17	0,09	1,24	0,06
Biogenic Amins	**Histamine**	0,61	0,03	0,60	0,09	0,63	0,04	0,8038	0,0463
Biogenic Amins	**Ac-Orn**	0,3	0,0	0,2	0,0	0,3	0,0	0,4	0,0
Biogenic Amins	**Spermine**	0,2	0,0	0,2	0,0	0,2	0,0	0,3	0,0
Biogenic Amins	**PEA**	0,22	0,01	0,23	0,05	0,20	0,01	0,37	0,07
Biogenic Amins	**DOPA**	0,06	0,01	0,09	0,01	0,08	0,01	0,11	0,02
Biogenic Amins	**Kynurenine**	0,04	0,00	0,04	0,00	0,05	0,00	0,06	0,00
Acylcarnitines	**C0 (free Carnitine)**	15,199	2,05	7,885	1,36	17,298	2,59	20,746	3,41
Acylcarnitines	**C2 (AcylCar)**	3,370	0,23	2,879	0,33	3,565	0,37	4,196	0,41
Acylcarnitines	**C3-DC (C4-OH)**	0,245	0,02	0,173	0,02	0,286	0,03	0,350	0,038
Acylcarnitines	**C6 (C4:1-DC)**	0,231	0,010	0,203	0,017	0,229	0,013	0,302	0,018
Acylcarnitines	**C5-M-DC**	0,201	0,008	0,251	0,032	0,240	0,010	0,377	0,044
Acylcarnitines	**C4**	0,179	0,009	0,132	0,007	0,166	0,010	0,206	0,020
Acylcarnitines	**C5-OH (C3-DC-M)**	0,160	0,009	0,126	0,004	0,188	0,015	0,233	0,018
Acylcarnitines	**C5-DC (C6-OH)**	0,151	0,007	0,118	0,005	0,174	0,008	0,243	0,022
Acylcarnitines	**C9**	0,107	0,005	0,091	0,012	0,114	0,013	0,161	0,018
Acylcarnitines	**C8**	0,087	0,004	0,081	0,009	0,094	0,003	0,123	0,006
Acylcarnitines	**C7-DC**	0,081	0,010	0,076	0,012	0,158	0,013	0,145	0,011
Acylcarnitines	**C10**	0,068	0,003	0,071	0,007	0,090	0,003	0,110	0,006
Acylcarnitines	**C3**	0,064	0,005	0,046	0,003	0,070	0,006	0,086	0,008
Acylcarnitines	**C12**	0,035	0,003	0,036	0,006	0,050	0,002	0,066	0,005
Acylcarnitines	**C4:1**	0,031	0,002	0,031	0,004	0,035	0,001	0,051	0,005
Acylcarnitines	**C3-OH**	0,031	0,003	0,017	0,002	0,032	0,003	0,078	0,008
Acylcarnitines	**C5:1-DC**	0,030	0,001	0,029	0,002	0,034	0,001	0,046	0,003
Acylcarnitines	**C6:1**	0,029	0,002	0,029	0,004	0,033	0,002	0,047	0,004
Acylcarnitines	**C3:1**	0,016	0,001	0,018	0,002	0,022	0,001	0,026	0,003
Acylcarnitines	**C14:2-OH**	0,008	0,001	0,009	0,002	0,015	0,003	0,019	0,005
Acylcarnitines	**C14:2**	0,008	0,001	0,009	0,002	0,011	0,001	0,012	0,001
Acylcarnitines	**C14:1**	0,007	0,000	0,008	0,001	0,009	0,001	0,012	0,001
Acylcarnitines	**C16**	0,007	0,001	0,008	0,001	0,010	0,000	0,011	0,001
Acylcarnitines	**C18**	0,005	0,001	0,005	0,001	0,009	0,001	0,010	0,002
Lysophosphatidy	**lysoPC a C18:0**	0,335	0,020	0,333	0,023	0,495	0,036	0,445	0,044
Lysophosphatidy	**lysoPC a C16:0**	0,248	0,015	0,262	0,036	0,439	0,036	0,322	0,044
Lysophosphatidy	**lysoPC a C28:0**	0,077	0,008	0,090	0,023	0,058	0,009	0,089	0,018
Lysophosphatidy	**lysoPC a C18:2**	0,057	0,004	0,061	0,006	0,100	0,008	0,078	0,010
Lysophosphatidy	**lysoPC a C18:1**	0,037	0,002	0,044	0,003	0,060	0,004	0,053	0,004
Lysophosphatidy	**lysoPC a C20:4**	0,033	0,002	0,042	0,002	0,035	0,002	0,052	0,010
Lysophosphatidy	**lysoPC a C17:0**	0,024	0,002	0,021	0,001	0,027	0,002	0,027	0,002
Lysophosphatidy	**lysoPC a C28:1**	0,009	0,001	0,011	0,001	0,011	0,002	0,012	0,001
Lysophosphatidy	**lysoPC a C26:1**	0,007	0,001	0,007	0,001	0,007	0,000	0,009	0,001

		WT		FGF21 KO		dKO		UCP1 KO	
		Mean	SE	Mean	SE	Mean	SE	Mean	SE
Phosphatidylcholines	**PC ae C38:6**	34,65	2,19	37,47	5,46	35,24	1,54	38,75	4,38
Phosphatidylcholines	**PC ae C38:5**	6,35	0,44	7,56	1,28	5,20	0,28	6,53	1,17
Phosphatidylcholines	**PC aa C38:0**	3,30	0,22	3,64	0,51	3,66	0,25	4,02	0,48
Phosphatidylcholines	**PC ae C36:4**	2,83	0,19	3,52	0,62	2,51	0,17	3,55	0,76
Phosphatidylcholines	**PC aa C40:5**	2,20	0,20	2,25	0,46	1,18	0,10	1,38	0,20
Phosphatidylcholines	**PC ae C40:6**	1,97	0,17	2,02	0,29	1,85	0,15	2,13	0,29
Phosphatidylcholines	**PC aa C36:0**	1,66	0,10	2,00	0,33	1,77	0,11	2,13	0,30
Phosphatidylcholines	**PC ae C36:5**	1,55	0,09	2,06	0,41	1,60	0,11	2,19	0,47
Phosphatidylcholines	**PC ae C34:1**	1,20	0,12	1,15	0,24	0,95	0,10	0,94	0,14
Phosphatidylcholines	**PC aa C38:5**	0,906	0,066	1,076	0,239	0,735	0,058	0,930	0,160
Phosphatidylcholines	**PC aa C38:1**	0,822	0,042	0,989	0,123	0,977	0,063	1,112	0,165
Phosphatidylcholines	**PC ae C34:2**	0,775	0,061	0,835	0,165	0,674	0,065	0,703	0,101
Phosphatidylcholines	**PC ae C38:4**	0,768	0,053	0,967	0,196	0,734	0,038	0,995	0,206
Phosphatidylcholines	**PC aa C36:6**	0,548	0,033	0,627	0,103	0,587	0,040	0,623	0,083
Phosphatidylcholines	**PC aa C40:4**	0,540	0,039	0,720	0,168	0,444	0,025	0,691	0,173
Phosphatidylcholines	**PC aa C38:3**	0,483	0,042	0,584	0,126	0,255	0,024	0,358	0,069
Phosphatidylcholines	**PC ae C32:1**	0,385	0,040	0,432	0,094	0,304	0,044	0,318	0,054
Phosphatidylcholines	**PC ae C36:3**	0,383	0,029	0,399	0,076	0,337	0,021	0,361	0,052
Phosphatidylcholines	**PC aa C38:6**	0,308	0,020	0,354	0,059	0,313	0,023	0,351	0,053
Phosphatidylcholines	**PC ae C30:0**	0,265	0,024	0,302	0,069	0,205	0,025	0,245	0,043
Phosphatidylcholines	**PC ae C40:5**	0,251	0,022	0,274	0,046	0,194	0,010	0,249	0,047
Phosphatidylcholines	**PC aa C40:6**	0,250	0,017	0,266	0,050	0,185	0,011	0,232	0,026
Phosphatidylcholines	**PC aa C38:4**	0,238	0,016	0,321	0,072	0,177	0,011	0,251	0,056
Phosphatidylcholines	**PC aa C36:1**	0,223	0,016	0,248	0,047	0,167	0,012	0,197	0,027
Phosphatidylcholines	**PC aa C36:3**	0,204	0,014	0,288	0,079	0,153	0,011	0,244	0,059
Phosphatidylcholines	**PC aa C42:4**	0,171	0,013	0,197	0,040	0,125	0,010	0,172	0,029
Phosphatidylcholines	**PC ae C36:2**	0,149	0,013	0,152	0,024	0,139	0,010	0,149	0,020
Phosphatidylcholines	**PC aa C36:5**	0,125	0,007	0,156	0,031	0,115	0,006	0,146	0,030
Phosphatidylcholines	**PC aa C34:1**	0,108	0,007	0,135	0,027	0,101	0,009	0,119	0,019
Phosphatidylcholines	**PC ae C34:0**	0,103	0,010	0,096	0,020	0,078	0,006	0,089	0,014
Phosphatidylcholines	**PC aa C36:4**	0,098	0,006	0,135	0,029	0,097	0,005	0,136	0,031
Phosphatidylcholines	**PC aa C32:0**	0,094	0,007	0,109	0,019	0,085	0,006	0,103	0,015
Phosphatidylcholines	**PC ae C34:3**	0,094	0,006	0,120	0,023	0,095	0,009	0,126	0,026
Phosphatidylcholines	**PC ae C38:0**	0,077	0,005	0,087	0,012	0,085	0,006	0,096	0,012
Phosphatidylcholines	**PC aa C42:5**	0,076	0,009	0,073	0,012	0,031	0,002	0,042	0,006
Phosphatidylcholines	**PC aa C40:3**	0,072	0,005	0,103	0,029	0,054	0,006	0,090	0,022
Phosphatidylcholines	**PC aa C42:6**	0,072	0,006	0,070	0,016	0,036	0,003	0,055	0,006
Phosphatidylcholines	**PC aa C36:2**	0,069	0,004	0,083	0,016	0,066	0,007	0,081	0,012
Phosphatidylcholines	**PC ae C36:1**	0,067	0,006	0,071	0,012	0,060	0,005	0,069	0,009
Phosphatidylcholines	**PC ae C38:3**	0,054	0,004	0,059	0,012	0,043	0,002	0,055	0,010
Phosphatidylcholines	**PC aa C34:2**	0,052	0,003	0,059	0,011	0,061	0,009	0,062	0,008
Phosphatidylcholines	**PC ae C40:4**	0,049	0,004	0,057	0,010	0,048	0,004	0,061	0,009
Phosphatidylcholines	**PC ae C32:2**	0,047	0,003	0,061	0,011	0,050	0,005	0,064	0,013
Phosphatidylcholines	**PC aa C34:4**	0,033	0,002	0,042	0,008	0,032	0,002	0,046	0,011
Phosphatidylcholines	**PC aa C32:1**	0,032	0,003	0,035	0,007	0,027	0,003	0,030	0,005
Phosphatidylcholines	**PC aa C40:2**	0,032	0,002	0,045	0,012	0,033	0,002	0,046	0,010
Phosphatidylcholines	**PC ae C30:1**	0,015	0,002	0,019	0,003	0,014	0,002	0,017	0,004
Phosphatidylcholines	**PC ae C38:2**	0,012	0,001	0,013	0,002	0,013	0,001	0,016	0,003
Phosphatidylcholines	**PC ae C38:1**	0,012	0,001	0,014	0,002	0,013	0,001	0,016	0,002
Phosphatidylcholines	**PC aa C34:3**	0,010	0,001	0,014	0,003	0,011	0,001	0,013	0,002
Phosphatidylcholines	**PC aa C42:0**	0,010	0,001	0,008	0,001	0,010	0,001	0,015	0,002
Phosphatidylcholines	**PC ae C40:3**	0,010	0,001	0,014	0,003	0,009	0,001	0,014	0,003
Phosphatidylcholines	**PC aa C32:2**	0,009	0,001	0,011	0,002	0,009	0,001	0,010	0,001
Phosphatidylcholines	**PC ae C40:1**	0,008	0,001	0,009	0,002	0,008	0,000	0,010	0,001
Phosphatidylcholines	**PC ae C42:2**	0,008	0,001	0,009	0,002	0,008	0,001	0,009	0,001
Phosphatidylcholines	**PC aa C28:1**	0,007	0,000	0,009	0,001	0,007	0,000	0,010	0,001
Phosphatidylcholines	**PC ae C42:3**	0,006	0,000	0,008	0,002	0,006	0,001	0,009	0,001
Phosphatidylcholines	**PC ae C40:2**	0,004	0,000	0,006	0,001	0,004	0,000	0,006	0,001
Phosphatidylcholines	**PC aa C42:1**	0,003	0,000	0,003	0,001	0,004	0,000	0,004	0,001
Phosphatidylcholines	**PC aa C32:3**	0,003	0,000	0,003	0,001	0,003	0,000	0,003	0,000
Phosphatidylcholines	**PC aa C30:2**	0,001	0,001	0,002	0,001	0,002	0,000	0,002	0,001
Sphingomyelins	**SM C16:0**	0,374	0,024	0,472	0,036	0,400	0,041	0,448	0,048
Sphingomyelins	**SM C24:0**	0,187	0,008	0,239	0,030	0,199	0,010	0,249	0,045
Sphingomyelins	**SM C16:1**	0,118	0,007	0,142	0,016	0,123	0,007	0,125	0,012
Sphingomyelins	**SM C24:1**	0,032	0,006	0,041	0,004	0,061	0,006	0,078	0,011
Sphingomyelins	**SM (OH) C22:1**	0,018	0,002	0,026	0,004	0,017	0,002	0,023	0,003
Sphingomyelins	**SM C18:0**	0,009	0,000	0,012	0,002	0,009	0,001	0,011	0,002
Sphingomyelins	**SM C18:1**	0,003	0,000	0,003	0,000	0,002	0,000	0,004	0,001
Sphingomyelins	**SM (OH) C16:1**	0,003	0,001	0,005	0,002	0,004	0,001	0,004	0,001
Sphingomyelins	**SM (OH) C24:1**	0,002	0,000	0,003	0,001	0,003	0,000	0,068	0,007

out of range: Taurine
under detection level: SDMA, c4-OH-Pro, Nitro-Tyr, C10:1, C5, C12:1, C12-DC, C5:1, C10:2, C14, C16:1, C16:2, C14:1-OH, C18:1, C18:2, C16:1-OH, C16-OH, C18:1-OH, lysoPC a C14:0 PC aa C26:0, lysoPC a C24:0, PC aa C30:0, lysoPC a C16:1, lysoPC a C20:3, lysoPC a C26:0, PC ae C42:5, PC ae C42:0, PC ae C36:0, PC ae C30:2, PC aa C42:2, PC aa C40:1, PC ae C30:2 PC aa C40:1, PC ae C44:4, PC ae C42:1, PC ae C44:5, PC ae C42:4, PC ae C44:6, PC ae C44:3, PC aa C24:0, SM C26:0, SM C26:1, SM (OH) C14:1, SM (OH) C22:2, SM C20:2, SM C22:3

Supplemental Table 2
Table of calculated net energy balance in each genotype.
Statistical significance was assumed at $p < 0.05$. Statistical differences between the four different genotypes are indicated by letters (n=5/6/6/5*, n= 11/6/12/9# WT/ FGF21 KO/ UCP1 KO/ dKO).

	WT	FGF21 KO	UCP1 KO	dKO
raw data				
Body mass (g)*	34.4 ± 2.3a	33.4 ± 1.3a	26.7 ± 0.4b	35.2 ± 0.8a
Energy intake (kJ/d)*	57.6 ± 1.5ab	57.6 ± 4.1ab	48.1 ± 2.0a	63.3 ± 4.2b
Metabolic rate (kJ/d)*	48.9 ± 0.6ab	47.6 ± 1.2ab	44.7 ± 1.4a	50.1 ± 1.0b
Feces energy (kJ/d)#	2.2 ± 0.03	2.9 ± 0.3	2.6 ± 0.2	2.6 ± 0.1
Urine energy (kJ/d)#	1.7 ± 0.2	1.6 ± 0.2	2.0 ± 0.2	2.3 ± 0.2
calculated energy balance				
Energy intake - energy outflow (kJ/d)	+ 4.8	+ 5.5	-1.2	+ 8.3
Calculated accumulated energy over 8 weeks (kJ)	268,8	308,0	-67,2	464,8
Calculated fat accumulation over 8 weeks (g)	6,9	7,9	-1,7	11,9
% energy partitioning (energy intake set 100%)				
Metabolic rate (%)	84,9	82,6	92,9	79,1
Feces energy (%)	3,8	5,0	5,4	4,1
Urine energy (%)	3,0	2,8	4,2	3,6
**Released energy (%)**	91,7	90,5	102,5	86,9

Supplemental Table 3
Related to Methods "Gene expression analysis"; Mouse qRT-PCR Primer Sequences

Gene	5'3' primer (for)	3'5' primer (rev)
Fgf21	GCTGCTGGAGGACGGTTACA	CACAGGTCCCCAGGATGTTG
Hprt	*CAGTCCCAGCGTCGTGATTA*	*AGCAAGTCTTTCAGTCCTGTC*

Supplemental methods:

Histology

Interscapular brown adipose tissue and inguinal white adipose tissue specimens were fixed in 4% paraformaldehyde (Roth chemicals) for 24hrs, embedded in low melting paraffin (Paraplast Plus®, Sigma Aldrich) for histological examination. Four µm-thick sections were cut using a rotary microtome (HSM55, Microm). Slides were mounted on superfrost glass slides (Menzel glass) dehydrated in increasing ethanol series and stained with hematoxylin and eosin (H&E) (Merck). Bright field images were obtained with the Keyence Microscope BZ-9000.
