# Supplementary material for: Endogenous FGF21-signaling controls paradoxical obesity resistance of UCP1-deficient mice
Source: Nat Commun. 2020 Jan 31;11:624. doi: 10.1038/s41467-019-14069-2 (PMC6994690; doi:10.1038/s41467-019-14069-2)
Supplement: Supplementary file 3 — Reporting Summary [file 41467_2019_14069_MOESM3_ESM.pdf]

nature research

Corresponding author(s): Martin Jastroch

Last updated by author(s): Nov 5, 2019

Reporting Summary

Nature Research wishes to improve the reproducibility of the work that we publish. This form provides structure for consistency and transparency in reporting. For further information on Nature Research policies, see Authors & Referees and the Editorial Policy Checklist .

Statistics

For all statistical analyses, confirm that the following items are present in the figure legend, table legend, main text, or Methods section.

n/a	Confirmed	
☐	☒	The exact sample size (*n*) for each experimental group/condition, given as a discrete number and unit of measurement
☐	☒	A statement on whether measurements were taken from distinct samples or whether the same sample was measured repeatedly
☐	☒	The statistical test(s) used AND whether they are one- or two-sided<br>*Only common tests should be described solely by name; describe more complex techniques in the Methods section.*
☐	☒	A description of all covariates tested
☐	☒	A description of any assumptions or corrections, such as tests of normality and adjustment for multiple comparisons
☐	☒	A full description of the statistical parameters including central tendency (e.g. means) or other basic estimates (e.g. regression coefficient) AND variation (e.g. standard deviation) or associated estimates of uncertainty (e.g. confidence intervals)
☐	☒	For null hypothesis testing, the test statistic (e.g. *F*, *t*, *r*) with confidence intervals, effect sizes, degrees of freedom and *P* value noted<br>*Give P values as exact values whenever suitable.*
☒	☐	For Bayesian analysis, information on the choice of priors and Markov chain Monte Carlo settings
☐	☒	For hierarchical and complex designs, identification of the appropriate level for tests and full reporting of outcomes
☒	☐	Estimates of effect sizes (e.g. Cohen's *d*, Pearson's *r*), indicating how they were calculated

Our web collection on statistics for biologists contains articles on many of the points above.

Software and code

Policy information about availability of computer code

Data collection	Data collection of metabolic cage analysis was performed using TSE PhenoMaster Software (TSE Systems GmbH, Bad Homburg, Germany). Data collection of qPCR were collected using the ViiA™ 7 Real-Time PCR System (Applied Biosystems). The energy content of the dry urine and feces residues were automatically calculated by the software of the IKA C7000 bomb calorimeter, Staufen, Germany. For ELISA/Serum analysis, the Pherastar spectrophotometer-fluorescence reader system (BMG Labtech) was used. For RNA seq analysis, The quality of the RNA was determined with the Agilent 2100 BioAnalyzer (RNA 6000 Nano Kit, Agilent). All samples had a RNA integrity number (RIN) value greater than 8. RNA libraries were assessed for quality and quantity with the Agilent 2100 BioAnalyzer and the Quant-iT PicoGreen dsDNA Assay Kit (Life Technologies). Strand specific RNA libraries were sequenced as 100 bp paired-end runs on an Illumina HiSeq4000 platform. Bacterial DNA was profiled by sequencing of the V4 region of the 16S rRNA gene on an Illumina MiSeq (llumina RTA v1.17.28; MCS v2.5) using 515F and 806R primers designed for dual indexing 60 and the V2 kit (2x250 bp paired-end reads).
Data analysis	Following codes were used to analyze data:<br>Transcriptomic Analysis: The STAR aligner* (v 2.4.2a) (Anders, S., et al.. HTSeq--a Python framework to work with high-throughput sequencing data. Bioinformatics 31, 166-169, 2015) with modified parameter settings (--twopassMode=Basic) was used for split-read alignment against the mouse genome assembly mm10 (GRCm38) and UCSC known Gene annotation. To quantify the number of reads mapping to annotated genes we used HTseq-count° (v0.6.0). Raw read counts were count files were normalized and DEG were estimated using R package DESeq2. All calculations were done using R version 3.4 and Matlab R2018a.<br><br>DNA extraction and 16S rRNA gene sequencing: Illumina paired-end reads were merged using PEAR (Zhang, J.,et al. PEAR: a fast and accurate Illumina Paired-End reAd mergeR. Bioinformatics 30, 614-620, 2014), and quality filtered to remove reads that had at least one base with a q-score lower than 20 and that were shorter than 220 nucleotides or longer than 350 nucleotides. Quality filtered reads were analyzed with the software package QIIME 62 (version 1.9.1). Sequences were clustered into operational taxonomic units (OTUs) at a 97% identity threshold using an open-reference OTU picking approach with UCLUST (Edgar, 2010) against the Greengenes reference database (DeSantis, T. Z. et al. Greengenes, a chimera-checked 16S rRNA gene database and workbench compatible with ARB. Applied and environmental microbiology 72, 5069-5072, 2006) (13_8 release). All sequences that failed to cluster when tested against the Greengenes database were used as input for picking OTUs de novo. Representative sequences for the OTUs were Greengenes reference

sequences or cluster seeds, and were taxonomically assigned using the Greengenes taxonomy and the Ribosomal Database Project Classifier (Wang, Q.et al. Bayesian classifier for rapid assignment of rRNA sequences into the new bacterial taxonomy. Applied and environmental microbiology 73, 5261-5267, 2007). Representative OTUs were aligned using PyNAST (Caporaso, J. G. et al. PyNAST: a flexible tool for aligning sequences to a template alignment. Bioinformatics 26, 266-267, 2010) and used to build a phylogenetic tree with FastTree (Price, M. et al. FastTree 2--approximately maximum-likelihood trees for large alignments. PloS one 5, e9490, 2010)., which was used to calculate α- and β-diversity of samples using Phylogenetic Diversity. Chimeric sequences were identified with ChimeraSlayer (Haas, B. J. et al. Chimeric 16S rRNA sequence formation and detection in Sanger and 454-pyrosequenced PCR amplicons. Genome research 21, 494-504, 2011). and excluded from all downstream analyses. Similarly, sequences that could not be aligned with PyNAST, singletons, sequences present in the blank extraction control and very low abundant sequences (relative abundance <0.005%) were also excluded. To correct for differences in sequencing depth, the same amount of sequences was randomly sub-sampled for each group of samples (rarefaction; maximum depth depending on sample group). A bootstrap version of Mann-Whitney-U test was used to compare the genotype-dependent abundance of OTUs at different taxonomical levels; significant differences were identified after correction for false discovery rate. Abundances higher than 1% are displayed on the genus level. QIIME was used to compute alpha diversity from rarefied OTU tables and to determine statistical significance at maximum rarefaction level by using a two-sample t-test and 999 Monte-Carlo permutations. Beta-diversity and weighted unifrac distance matrix were computed with QIIME and statistical significance of sample groupings was determined by adonis method and 999 permutations.

For manuscripts utilizing custom algorithms or software that are central to the research but not yet described in published literature, software must be made available to editors/reviewers. We strongly encourage code deposition in a community repository (e.g. GitHub). See the Nature Research guidelines for submitting code & software for further information.

Data

Policy information about availability of data

All manuscripts must include a data availability statement. This statement should provide the following information, where applicable:

- Accession codes, unique identifiers, or web links for publicly available datasets
- A list of figures that have associated raw data
- A description of any restrictions on data availability

Data availability statement has been provided.

Field-specific reporting

Please select the one below that is the best fit for your research. If you are not sure, read the appropriate sections before making your selection.

☒ Life sciences ☐ Behavioural & social sciences ☐ Ecological, evolutionary & environmental sciences

For a reference copy of the document with all sections, see nature.com/documents/nr-reporting-summary-flat.pdf

Life sciences study design

All studies must disclose on these points even when the disclosure is negative.

Sample size	Sample size estimation was based upon our own previous results in comparable studies, assuming to achieve 90% power at a significance level of 0.05.
Data exclusions	Of metabolic cage analysis, data were excluded from obvious technical error that would result in false data collection. Specifically, the food-hopper did not registrate data in one case, and water bottle leakage occured in two other cases. Non-responders during GTT's glucose admission were not included. Otherwise, no data were excluded from the analysis unless Grubbs test for outlier justified the exclusion of a significant outlier
Replication	Reproducibility of data was ensured by using independent mouse cohorts, showing reproducibility of genotype-dependent body weight progression. Each mouse was treated as independent biological sample.
Randomization	All experimental mice were randomized during data assessment.
Blinding	Complete blinding of the investigators was not possible with regard to diet and genotypes of the mice. Blinded data collection and/or analysis were performed in following experiments: all metabolic data of living mice and histological stainings. RNA sequencing data were collected blinded by using alphanumeric coding, post-analysis was performed non-blinded.

Reporting for specific materials, systems and methods

We require information from authors about some types of materials, experimental systems and methods used in many studies. Here, indicate whether each material, system or method listed is relevant to your study. If you are not sure if a list item applies to your research, read the appropriate section before selecting a response.

Materials & experimental systems

n/a	Involved in the study	
☒	☐	Antibodies
☒	☐	Eukaryotic cell lines
☒	☐	Palaeontology
☐	☒	Animals and other organisms
☒	☐	Human research participants
☒	☐	Clinical data

Methods

n/a	Involved in the study	
☒	☐	ChIP-seq
☒	☐	Flow cytometry
☒	☐	MRI-based neuroimaging

Animals and other organisms

Policy information about studies involving animals; ARRIVE guidelines recommended for reporting animal research

Laboratory animals	The experiments were performed in adult homozygous male WT, FGF21 KO, UCP1 KO and UCP1/FGF12 double KO (dKO) mice (genetic background C57BL/6J).
Wild animals	The study did not involve animals from the wild.
Field-collected samples	The study did not involve samples collected in the field
Ethics oversight	The animal welfare authorities of the local animal ethics committee of the state of Bavaria approved animal maintenance and experimental procedures in accordance with European guidelines.

Note that full information on the approval of the study protocol must also be provided in the manuscript.
